# Changes in the incidence and prevalence of ischemic stroke and associations with natural disasters: an ecological study in 193 countries

**DOI:** 10.1038/s41598-022-05288-7

**Published:** 2022-02-02

**Authors:** Kai-Sen Huang, Ding-Xiu He, Qianlan Tao, Yan-Yan Wang, Yong-Qiang Yang, Biao Zhang, Gang Mai, Debarati Guha-Sapir

**Affiliations:** 1 Department of Cardiology, People’s Hospital of Deyang City, Affiliated Hospital of Chengdu University of Traditional Chinese Medicine, Deyang, Sichuan China; 2 Department of Cardiology, The Affiliated Hospital of Southwest Medical University, Luzhou, Sichuan China; 3 Department of Respiratory and Critical Care Medicine, West China Hospital, Sichuan University, Chengdu, China; 4 Department of Emergency, People’s Hospital of Deyang City, Affiliated Hospital of Chengdu University of Traditional Chinese Medicine, Deyang, Sichuan China; 5 Chengdu Medical College, Chengdu, Sichuan China; 6 Center of Gerontology and Geriatrics and National Clinical Research Center for Geriatrics, West China Hospital, Sichuan University, Chengdu, China; 7 Department of Neurology, People’s Hospital of Deyang City, Affiliated Hospital of Chengdu University of Traditional Chinese Medicine, Deyang, Sichuan China; 8 Department of General Surgery, People’s Hospital of Deyang City, Affiliated Hospital of Chengdu University of Traditional Chinese Medicine, Deyang, Sichuan China; 9 Centre for Research on the Epidemiology of Disasters, Institute of Health and Society, University of Louvain, Brussels, Belgium

**Keywords:** Natural hazards, Cardiology

## Abstract

Epidemiological studies have indicated that natural disasters have important impacts on ischemic stroke. This study determined the associations between natural disasters and the incidence and prevalence of ischemic stroke at the global level. A 28-year ecological trend study was performed to estimate worldwide changes in the incidence and prevalence of ischemic stroke and their associations with natural disasters by analyzing data from 193 countries. Quantum geographic information system-based visualization and multivariable linear regression were used. Changes in the incidence and prevalence of ischemic stroke, as well as disaster occurrence, varied among the different regions over the past 28 years (*p* < 0.001). Multiple linear regression revealed an independent and positive association between disaster occurrence and the incidence of ischemic stroke in males, females and both sexes combined (standardized coefficients = 0.515, 0.470 and 0.483, *p* < 0.001); similar associations were found for the prevalence of ischemic stroke (standardized coefficients = 0.471, 0.417 and 0.438, *p* < 0.001). The incidence and prevalence of ischemic stroke changed significantly at the global level and were independently associated with natural disasters. Both males and females show common but different vulnerabilities to natural disasters. This evidence supports policy making and resource allocation for disaster response and disease burden reduction.

## Introduction

Ischemic stroke, defined as stroke due to brain ischemia resulting in an interruption or reduction in blood flow to a part of the brain, is a leading contributor to the global disease burden^1^. Unlike other established socioeconomic or demographic risk factors for ischemic stroke, such as smoking and hypertension, a natural disaster is defined by the World Health Organization (WHO)^2^ as an act of nature of such magnitude that it creates a catastrophic situation and has an immediate impact on the population, often resulting in the destruction of the physical, biological and social environment of those affected, creating short- and long-term impacts on public health. In recent decades, the study of the effects of natural disasters on stroke incidence, prevalence and mortality has become an emerging subfield within stroke epidemiology^2–9^.

To prevent an epidemic and improve public health, many epidemiological and clinical studies have been conducted to explore the association between natural disasters and ischemic stroke^2–7^. These studies found an increased risk of cerebrovascular events and mortality after a series of natural disasters, including earthquakes, extreme weather conditions, floods, viral pandemics, etc. Possible underlying mechanisms of this association may include environmental contamination exposure, emotional stress and lifestyle changes^10–12^. However, the previous studies have been somewhat heterogeneous in their study designs and populations. We still need solid evidence to confirm the independent association between the integrated impacts of all kinds of natural disasters and ischemic stroke at the global level.

Recently, the Global Burden of Disease (GBD) Study by the Institute for Health Metrics and Evaluation (IHME)^1^ published findings on worldwide country-specific incidence and prevalence of ischemic stroke from 1990 to 2017. We collected data on the impacts of natural disasters during the same period, quantified as disaster frequency (occurrence) and severity (casualties and total damage). The data on natural disasters were obtained from the Centre for Research on the Epidemiology of Disasters (CRED)^13^. Based on the above data, we conducted an ecological trend study to initially investigate the hypothesis that a natural disaster—as an environmental factor—is associated with the incidence and prevalence of ischemic stroke to promote appropriate adaptation measures and strategies for reducing the ischemic stroke burden.

## Results

**Figure 1:**
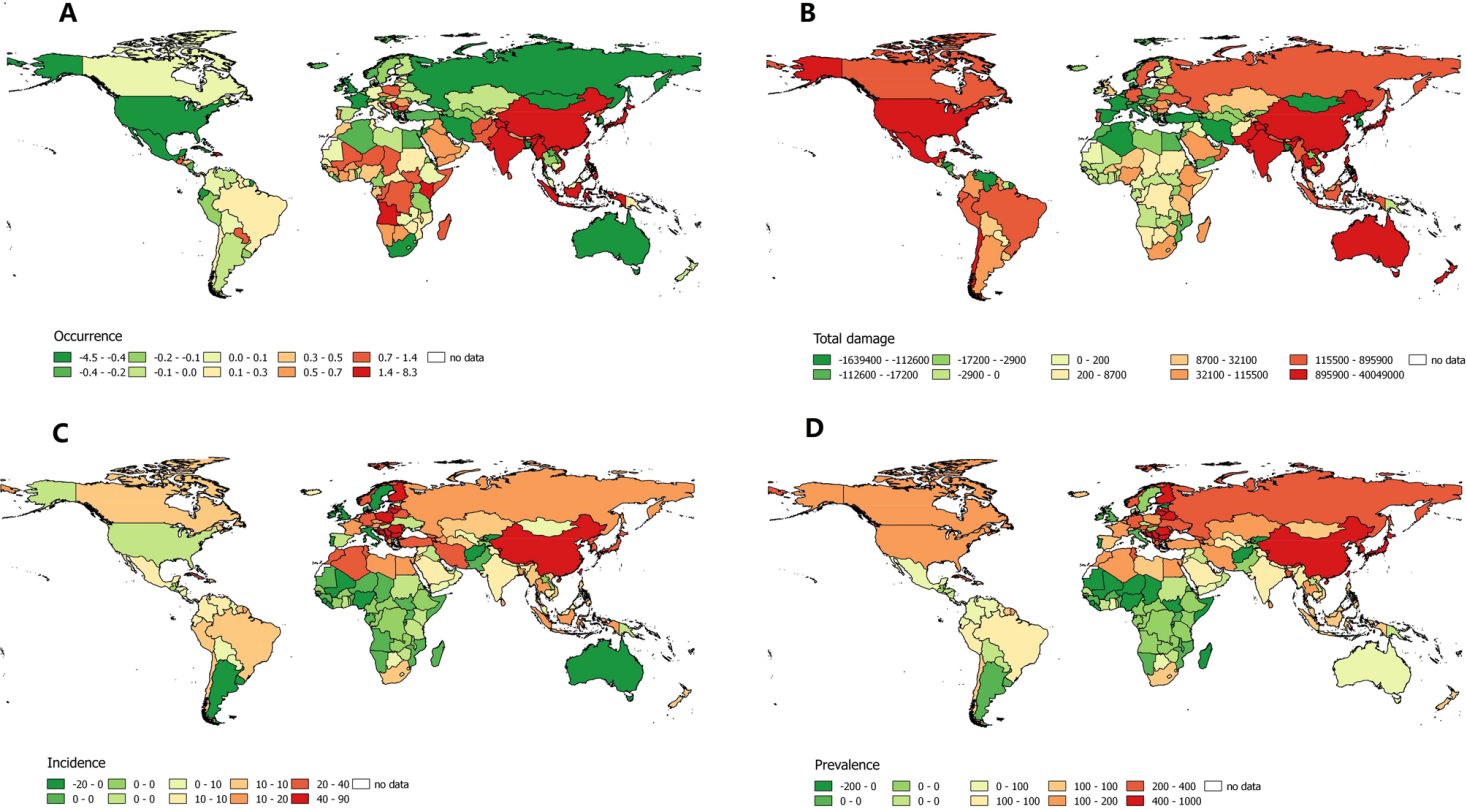
Map of trends in natural disaster occurrence, total damage, and incidence and prevalence of ischemic stroke during two periods (1990–2003 and 2004–2017) in 193 countries (generated by OSGeo2.6.1). (**A**) Trend in the occurrence of natural disasters per year. (**B**) Trend in total damage due to natural disasters, US$ 1000 per year. (**C**) Trend in the incidence of ischemic stroke in both sexes per 100,000 population per year. (**D**) Trend in the prevalence of ischemic stroke in both sexes per 100,000 population per year.

The descriptive statistics for natural disasters and ischemic stroke are summarized separately for the periods 1990–2003 and 2004–2017, and trends in both periods are presented (Table 1). The median values of natural disaster and ischemic stroke variables were also significantly different from each other (Wilcoxon signed-rank test; *p* < 0.05). Geographically, visual inspection indicated an increase in natural disaster occurrence, accompanied by an increased incidence and prevalence of ischemic stroke in densely populated developing regions (including East, South and Southeast Asia). Moreover, a decrease in occurrence was observed in developed regions (including North America and Western Europe), accompanied by a decreased incidence and prevalence of ischemic stroke (Fig. 1).

**Table 1:** Summary statistics of natural disaster impacts and incidence and prevalence of ischemic stroke during two periods in 193 countries.

	1990–2003	2004–2017	Trends	*P* value*
**Natural disaster**				
Occurrence, median (IQR)	0.86 (1.82)	1.13 (33.47)	0.12 (0.61)	0.000
Casualties, median (IQR)	4.29 × 10^2^ (6.38 × 10^3^)	2.06 × 10^2^ (2.05 × 10^3^)	− 20.43 (1.91 × 10^3^)	0.001
Total damage, median (IQR)	1.20 × 10^4^ (8.40 × 10^4^)	1.65 × 10^4^ (1.49 × 10^5^)	0 (4.74 × 10^4^)	0.019
**Incidence**				
Females	48.46 (47.86)	56.13 (57.24)	5.46 (13.58)	0.000
Males	47.96 (49.79)	55.36 (61.22)	5.87 (14.94)	0.000
Both	47.53 (50.62)	56.72 (55.47)	5.55 (14.42)	0.000
**Prevalence**				
Females	543.99 (529.70)	653.87 (671.26)	78.74 (160.11)	0.000
Males	518.07 (528.74)	608.93 (658.68)	67.62 (177.17)	0.000
Both	523.43 (499.89)	627.72 (616.71)	76.06 (159.95)	0.000

*Wilcoxon signed-rank test.

The results of the multivariable linear regression analysis for females are shown in Table 2. With all independent variables (natural disaster impacts and confounding variables) involved in regression weighted by population, natural disaster occurrence was independently and significantly associated with the incidence of ischemic stroke (standardized β = 0.47, *p* < 0.001) after adjusting for confounding variables. A considerable variance of 79.9% can be explained by this regression model for females. As a comparison, a variance of 73.2% can be explained by regression model with all confounding variables (Supplemental material Table 1). Consistently, a similar association between natural disaster occurrence and prevalence of ischemic stroke was found (standardized β = 0.417, *p* < 0.001), explaining 76.4% of the variance. As a comparison, a variance of 72.3% can be explained by regression model with all confounding variables (Supplemental material Table 1).

**Table 2:** Multivariable linear regression-derived coefficients for natural disaster and socioeconomic variables for the incidence and prevalence of ischemic stroke in females from 1990 to 2017 in 193 countries, weighted by population.

Factors	Incidence*			Prevalence^a^		
	Coefficient (95% CI)	Standardized coefficients	*P* value	Coefficient (95% CI)	Standardized coefficient	*P* value
**Natural disaster**						
Occurrence	2.810 ± 0.374	0.470	0.000	23.070 ± 3.700	0.417	0.000
Casualties (deaths + injuries)	NA^b^	− 0.065	0.157	NA	− 0.083	0.084
Total damage	NA	− 0.051	0.401	NA	− 0.100	0.130
Fat and meat consumption	NA	− 0.002	0.952	NA	0.007	0.845
Tobacco use	0.686 ± 0.200	0.120	0.001	6.544 ± 1.990	0.124	0.001
Alcohol consumption	0.350 ± 0.061	0.216	0.000	4.339 ± 0.611	0.289	0.000
Health expenditure	0.009 ± 0.001	0.268	0.000	0.125 ± 0.013	0.393	0.000
CO_2_ emissions	3.745 ± 0.846	0.276	0.000	38.953 ± 8.461	0.310	0.000
Trade (% of GDP)	0.104 ± 0.049	0.073	0.034	NA	0.061	0.097
Urban population (% of total)	0.587 ± 0.193	0.179	0.003	4.296 ± 1.919	0.142	0.026

*The stepwise method was used, R^2^ = 0.799, F = 103.811, *p* = 0.000.

^a^The stepwise method was used, R^2^ = 0.764, F = 99.377, *p* = 0.000.

^b^Not applicable for variables not included as predictors.

The results of the multivariable linear regression analysis for males are shown in Table 3. With all independent variables (natural disaster impacts and confounding variables) involved in regression weighted by population, occurrence remained independently and significantly associated with the incidence of ischemic stroke (standardized β = 0.515, *p* < 0.001) after adjusting for confounding variables. A considerable variance of 82.9% can be explained by this regression model for males. As a comparison, a variance of 75.4% can be explained by regression model with all confounding variables (Supplemental material Table 2). A similar association between natural disaster occurrence and the prevalence of ischemic stroke was found in this model (standardized β = 0.471, *p* < 0.001), explaining 79.5% of the variance. As a comparison, a variance of 72.9% can be explained by regression model with all confounding variables (Supplemental material Table 2).

**Table 3:** Multivariable linear regression-derived coefficients for natural disaster and socioeconomic variables for the incidence and prevalence of ischemic stroke in males from 1990 to 2017 in 193 countries, weighted by population.

Factors	Incidence*			Prevalence^a^		
	Coefficient (95% CI)	Standardized coefficient	*P* value	Coefficient (95% CI)	Standardized coefficient	*P* value
**Natural disaster**						
Occurrence	3.594 ± 0.434	0.515	0.000	27.710 ± 3.717	0.471	0.000
Casualties (deaths + injuries)	− 1.816 ± 0.702 × 10^−4^	− 0.109	0.010	NA	− 0.073	0.114
Total damage	− 4.717 ± 1.627 × 10^−7^	− 0.168	0.004	NA	− 0.101	0.103
Fat and meat consumption	NA^b^	0.025	0.443	NA	0.012	0.733
Tobacco use	0.551 ± 0.215	0.083	0.011	6.386 ± 1.984	0.114	0.002
Alcohol consumption	0.476 ± 0.067	0.252	0.000	3.781 ± 0.610	0.237	0.000
Health expenditure	0.020 ± 0.002	0.493	0.000	0.182 ± 0.013	0.537	0.000
CO_2_ emissions	3.613 ± 0.929	0.228	0.000	37.711 ± 8.410	0.228	0.000
Trade (% of GDP)	NA	0.050	0.124	1.096 ± 0.482	0.078	0.024
Urban population (% of total)	0.795 ± 0.214	0.208	0.000	5.117 ± 1.916	0.159	0.008

*The stepwise method was used, R^2^ = 0.829, F = 110.674, *p* = 0.000.

^a^The stepwise method was used, R^2^ = 0.795, F = 101.269, *p* = 0.000.

^b^Not applicable for variables not included as predictors.

The results of the multivariable linear regression analysis for both sexes are shown in Table 4. With all independent variables (natural disaster impacts and confounding variables) involved in regression weighted by population, natural disaster occurrence was unsurprisingly associated with the incidence of ischemic stroke (standardized β = 0.483, *p* < 0.001) after adjusting for confounding variables. A considerable variance of 81.8% can be explained by this regression model. As a comparison, a variance of 75.1% can be explained by regression model with all confounding variables (Supplemental material Table 3).A similar association between natural disaster occurrence and the prevalence of ischemic stroke was found in this model (standardized β = 0.438, *p* < 0.001), explaining 78.6% of the variance. As a comparison, a variance of 72.7% can be explained by regression model with all confounding variables (Supplemental material Table 3).

**Table 4:** Multivariable linear regression-derived coefficients for natural disaster and socioeconomic variables for the incidence and prevalence of ischemic stroke in both sexes from 1990 to 2017 in 193 countries, weighted by population.

Factors	Incidence*			Prevalence^a^		
	Coefficient (95% CI)	Standardized coefficient	*P* value	Coefficient (95% CI)	Standardized coefficient	*P* value
**Natural disaster**						
Occurrence	3.119 ± 0.384	0.483	0.000	24.891 ± 3.665	0.438	0.000
Casualties (deaths + injuries)	NA^b^	0.065	0.131	NA	− 0.072	0.127
Total damage	NA	− 0.095	0.102	NA	− 0.101	0.108
Fat and meat consumption	NA	− 0.005	0.876	NA	0.006	0.867
Tobacco use	0.622 ± 0.205	0.101	0.003	6.651 ± 1.957	0.123	0.001
Alcohol consumption	0.407 ± 0.063	0.233	0.000	4.147 ± 0.602	0.270	0.000
Health expenditure	0.012 ± 0.001	0.318	0.000	0.153 ± 0.013	0.470	0.000
CO_2_ emissions	3.955 ± 0.846	0.270	0.000	38.383 ± 8.293	0.298	0.000
Trade (% of GDP)	0.105 ± 0.050	0.069	0.036	0.952 ± 0.476	0.070	0.047
Urban population (% of total)	0.652 ± 0.198	0.184	0.001	4.861 ± 1.889	0.156	0.011

*The stepwise method was used, R^2^ = 0.818, F = 117.759, *p* = 0.000.

^a^The stepwise method was used, R^2^ = 0.786, F = 96.085, *p* = 0.000.

^b^Not applicable for variables not included as predictors.

## Discussion

### Main findings

The incidence and prevalence of ischemic stroke have increased in the regions of East, South and Southeast Asia and decreased in the regions of North America and Western Europe over the last 28 years. A significant and positive association between natural disasters and ischemic stroke was found in our study. Both males and females were equally vulnerable to ischemic stroke following a natural disaster. Our findings indicated that a natural disaster has a significant impact on ischemic stroke on the global scale.

### Comparisons with other studies

Ischemic stroke is a leading contributor to the global disease burden, accounting for 5.83% of global deaths and 2.51% of disability-adjusted life years (DALYs)^1^. Under the current risk assessment framework, 87.9% of ischemic stroke DALYs can be attributed to established risk factors (such as hypertension, high glucose, high total cholesterol and smoking), with the remaining burden due to unknown or unmeasured risk factors^14^. Over the last several years, an increasing number of epidemiological and clinical studies have found an increased risk of ischemic stroke after a series of natural disasters.

Sokejima et al.^15^ found that after the Hanshin-Awaji Earthquake in Japan, the incidence of ischemic and hemorrhagic stroke increased in the first year, and the increase was associated with seismic intensity in a dose–response manner after adjusting for age, sex, and income. Similar findings were reported by Omama et al.^16^ after the Great East Japan Earthquake and Tsunami in 2011; they found that the standard incidence ratios for ischemic stroke were 1.51 (1.19–1.88) for men, 1.35 (1.06–1.64) for subjects aged ≥ 75 years, and 1.35 (1.06–1.64) for people in high-flood areas. In addition to geological disasters such as earthquakes, climate change-related natural disasters (including biological, hydrological, meteorological and climatological disasters) have been found to be related to ischemic stroke. Chen et al.^8^ reported significantly increased ischemic and hemorrhagic stroke rates 3 weeks after flood events, with the rates increasing in a dose-dependent manner. A systematic review of 20 studies^17^ concluded a positive relationship between a 1 °C change and the occurrence of major adverse cerebrovascular events, with 1.1% (95% confidence intervals (CI), 0.6–1.7) and 1.2% (95% CI, 0.8–1.6) increases for hot and cold effects, respectively. This association between ambient temperature and ischemic stroke has been confirmed in recent studies^3–5^. Li et al.^18^ projected that in 2080, the total number of temperature-related, ischemic stroke-related deaths will have increased by approximately 100% compared with that in the 1980s. A recent study noted the disturbing possibility that coronavirus disease 2019 (COVID-19), as a type of biological disaster, could increase the ischemic stroke incidence^2^. However, the previous studies have been somewhat heterogeneous in their designs and populations, with few adjustments for confounding variables, although the results from diverse epidemiological studies have been consistent. We need additional evidence to confirm the independent associations between the integrated impacts of all kinds of natural disasters and ischemic stroke at the global level.

### Interpretation

The multivariable linear regression analyses with natural disasters and confounding variables involved indicate better predictive abilities than analyses with only confounding variables involved. After adjusting for confounding variables, the multivariable linear regression analysis in our study suggested that each single-incident increase in the annual natural disaster occurrence was associated with increases in the incidence of ischemic stroke of approximately 2.810 per 100,000 for females, 3.594 per 100,000 for males and 3.119 per 100,000 for both sexes. Each single-incident increase in the annual natural disaster occurrence was associated with increases in prevalence of ischemic stroke of approximately 23.070 per 100,000 for females, 27.710 per 100,000 for males and 24.891 per 100,000 for both sexes. Moreover, considerable variances of approximately 80% can be explained by our regression models. Furthermore, it seems that males are slightly more vunerable to natural disaster than females in incidence(standardized coefficients = 0.515 vs. 0.470) and prevalence (standardized coefficients = 0. 471 vs. 0. 417). However, our study showed that other measures of the severity of natural disasters, including casualties and total damage, were not significantly associated with ischemic stroke. This finding suggests that the impact of natural disasters extends far beyond the apparently directly affected population.

Although the association between natural disaster and ischemic stroke has been confirmed by recent studies, the mechanism of this association is still unclear because of the unpredictability of natural disasters. Several explanations can be proposed, as follows: (a) disasters directly impacts physical health, stimulating conditions such as autonomic system imbalance, hemodynamic disorders, sympathetic and renin angiotensin system activation, oxidative stress and inflammation, or mental disorders^19–21^; (b) disaster victims are exposed to risk factors; an increasing number of studies have reported that catastrophic natural disasters are related to increased exposure to established risk factors, such as hypertension, increased body mass index (BMI), smoking, alcohol abuse, high blood glucose and high-sodium diets^11,22^; and (c) access to medical services is hindered, as natural disasters often result in the destruction of infrastructure and social environments, creating difficulties in accessing medical services^23^.

### Strengths and limitations

Several strengths of our study merit mention. First, our study provides the first global-level evidence from 193 countries using two indicators: incidence and prevalence. Second, unlike previous observational studies, a series of relevant confounding variables were included and adjusted in the same set in the multivariable linear regression analysis, ensuring the reliability of our study. Third, we studied the ecological trends of natural disasters and ischemic stroke in the whole population, thus avoiding the disadvantages of previous studies, which include difficulties in identifying disaster-affected and control populations, especially from a psychosocial perspective. Fourth, the similarity of the results of the subgroup analyses (males, females and both sexes) and different methods (visualization and regression analyses) confirmed the robustness of our findings.

Our study also has several limitations that need to be mentioned. The main limitation is intrinsic limitation of the ecological study design (conceptualized as the ecological fallacy), meaning that the results obtained from populations cannot be extrapolated to individuals. Another limitation is that, as indicated in the inclusion criteria, the natural disasters included in our study were extremely destructive. Whether natural disasters of lesser severity have similar impacts on ischemic stroke is unknown. In addition, most of the confounding variables included in our study were population-related factors. Individual intermediate factors (e.g., blood pressure, blood glucose, BMI) could be included in the future if data are available. Finally, our study reflects the associations among trends in variables over the long term, leaving short-term associations undefined.

### Implications of this study

Although an increasing number of studies have suggested an increased risk of ischemic stroke following all kinds of natural disasters, the value of our study lies in the fact that it is imperative to increase awareness of the association between the occurrence of natural disasters and ischemic stroke. In addition, although the occurrence of natural disasters may be nonmodifiable and ubiquitous, adaptation strategies, such as environmental restoration, psychological interventions, lifestyle improvements and infrastructure reconstruction, can be developed and promoted to aid natural disaster response and to reduce the risk of ischemic stroke. To achieve these goals, additional detailed data on natural disaster exposures, subsequent health effects and potential mechanisms are particularly needed.

## Conclusions

The changes in the incidence and prevalence of ischemic stroke varied among different regions over the last 28 years. Our ecological study confirmed and quantified the association between the occurrence of natural disasters and ischemic stroke in terms of incidence and prevalence in 193 countries. Both males and females show common but different vulnerabilities to ischemic stroke following natural disasters. Our study provides evidence to support policy making and resource allocation for disaster response and reduce the ischemic stroke burden.

## Methods

### Study design

An ecological trend design was used to assess the associations between natural disasters and ischemic stroke in a population consisting of all individuals in 193 countries. The unit of observation was a single year during the observation period from 1990 to 2017. The 28 years with available country-specific incidence and prevalence rates of ischemic stroke were divided into two periods, 1990–2003 and 2004–2017.

### Patient and public involvement

Since our study was a population-based ecological trend study using data accessible from open sources and no identification of individuals was needed for the recruitment or conduct of the study, informed consent from participants and approval from the ethics research committee were waived.

### Variable definition and data collection

The analysis of variable trends relied on original data from open sources, including the CRED, the GBD Study, and the World Bank and the Food and Agriculture Organization of the United Nations (FAO). The annual means of each variable were calculated with original data from two periods (1990–2003 and 2004–2017). The trends of the variables in the two periods were calculated based on the following formula:

Variable trend = variable mean (2004–2017)—variable mean (1990–2003).

Trends were replaced by 0 for unavailable variables. All the original data for each variable were collected from the same 193 countries. A list of the included countries is provided in the supplemental information. Countries with any unavailable data were excluded. The data that support the findings of this study are available from the corresponding author upon reasonable request.

#### Independent variables: impacts of natural disaster

The impacts of natural disasters were quantified as occurrence, casualties and total damage; this information was obtained from the Emergency Events Database^10^ (EM-DAT) of the CRED—a WHO Collaborating Centre since 1980. The EM-DAT contains essential core data on the annual occurrence and effects of natural disasters worldwide from 1900 to the present. Natural disasters encompass all the main categories of catastrophic events, including geophysical, meteorological, hydrological, climatological, biological and extraterrestrial disasters. For a disaster to be entered into the EM-DAT database, at least one of the following criteria must be met: (a) ten or more people died, (b) one hundred or more people were affected, (c) of a state of emergency declaration was made, or (d) international assistance was officially requested. The variables of natural disaster exposure were defined as follows:

*Occurrence (per year)* The average annual number of natural disasters in each country during a certain period.

*Casualties (per 100,000 per year)* The average annual number of deaths and injuries due to a natural disaster during a certain period.

*Total damage (US$ 1000 per year)* The value of all damage and economic losses directly or indirectly related to the disaster. This information includes a breakdown of the figures by sector: social, infrastructure, production, environment and other (when available).

#### Independent variables: confounding variables

Data on five other variables considered confounding factors were collected from two sources: the Word Bank and the FAO. The data of four variables were obtained from the World Bank^24^: health expenditure per capita, purchasing power parity (PPP) (current international $), CO_2_ emissions (metric tons per capita), urban population (%), and trade (% of GDP). Additionally, the population data of each country was obtained from the World Bank as a weighting factor for multivariable regression. The data for three variables were obtained from the FAO^25^: tobacco consumption per capita (kg), alcohol consumption per capita (kg), and fat and meat consumption per capita (kg).

#### Dependent variables: incidence and prevalence of ischemic stroke

The GBD Study provided the source data on the incidence and prevalence rates of ischemic stroke (International Classification of Diseases Tenth Edition, Clinical Modification ([ICD-10-CM] code I63), which are essential for disease burden assessment and prevention planning. The GBD Study provides data on incidence and prevalence rates as health outcomes from 1990 to 2017 for males, females and both sexes combined. These dependent variables were defined as follows:

*Incidence (per 100,000 per year)* annual new cases per 100,000 population (all ages) in that year.

*Prevalence (per 100,000 per year)* annual total cases per 100,000 population (all ages) in that year.

### Statistical analyses

Statistical analyses of 193 countries were conducted using IBM SPSS version 22 (IBM, Armonk, New York, USA), and a *p* value < 0.05 was considered to be statistically significant. Continuous variables are presented as medians and quartiles for nonnormally distributed data. The methods of statistical analysis of this study are refer to previous studies^26,27^.

The Wilcoxon signed rank test was used to assess natural disaster impacts and ischemic stroke variables with the same distribution. The correlations between trends of natural disaster impacts and ischemic stroke were visualized by a world map of trends with Quantum Geographic Information Systems (QGIS) (OSGeo2.6.1, Beaverton, OR, USA).

Multivariable linear regression was used to investigate the associations between the trends in the dependent variables (incidence and prevalence of ischemic stroke in males, females and both sexes) and independent variables (natural disaster impacts and confounding variables), with the criteria for entry and removal set at 0.05 and 0.1, respectively. Firstly, all confounding variables were be involved in multivariable linear regression with the backward stepwise methodology (Wald) and weighting according to the average population size from 1990 to 2017. Coefficients with 95% confidence intervals (95% CIs) and p values are reported for the variables included. Additionally, standardized coefficients and R^2^ were calculated to evaluate the size of the effect for all variables and predictive abilities. Secondly, all independent variables (natural disaster impacts and confounding variables) were be involved in multivariable linear regression with same procedure. Coefficients with 95% confidence intervals (95% CIs), standardized coefficients and R^2^ are also reported to compare with counterparts in the first step. Subgroup analyses (males, females and both sexes) and different methods (visualization and regression analyses) were used to confirm the robustness of the findings.

## Supplementary Information

Supplementary Information 1.

Supplementary Information 2.

Supplementary Tables.

## Data Availability

Data for this article can be found online. EMDAT, https://www.emdat.be/emdat_db/. FAO, http://www.fao.org/faostat/en/#data.IHME,http://ghdx.healthdata.org/gbd-results-tool. The World Bank, https://data.worldbank.org/indicator?tab=all.
