# Supplementary material for: Changes in the incidence and prevalence of ischemic stroke and associations with natural disasters: an ecological study in 193 countries
Source: Sci Rep. 2022 Feb 2;12:1808. doi: 10.1038/s41598-022-05288-7 (PMC8810883; doi:10.1038/s41598-022-05288-7)
Supplement: Supplementary file 2 — Supplementary Information 2. [file 41598_2022_5288_MOESM2_ESM.pdf]

country	Occurrence	Total damage	casualty	Alcohol	FVFM	Tobacco	health expen	CO2	trade	urbanpopulation	Incidencetotal	Prevalencetotal	population
Af	1.395238095	5531.666667	2807.657143	0.118285714	0.347585322	2.140599901	56.32070933	0.079380191	-30.51364042	2.610038596	-15.90468653	-94.25649045	2.47E+07
Albania	0.49047619	-1145.133333	-0.404761905	15.95928571	5.154238048	-3.542674446	257.1017042	0.199284549	23.2176162	14.35853333	37.90760123	357.7919563	3037644.793
Algeria	-0.40952381	-304527.1286	-1001.847619	1.490714286	55.76654841	1.351912772	342.468044	0.17904983	16.27309555	12.88248772	20.57882323	176.3034144	3.33E+07
American Samoa	0.128571429	6428.571429	5.623809524	0	0	0	0	0	-23.30852146	3.554982456	10.8769983	124.8384537	55596.41379
Angola	1.442857143	-580.952381	581.3380952	40.23671429	5.132585241	3.633514644	59.16314814	0.673974568	-36.07621438	18.05548772	-2.681579737	-7.648064231	1.98E+07
Antigua and Barbuda	-0.085714286	-14636.19048	-15.21904762	2.915571429	4.844789789	-3.220834161	361.2063299	1.272031324	-32.07949596	-7.345670175	-0.262064757	24.64464242	80167.65517
Argentina	-0.061904762	42288.80952	-12.38571429	-6.228571429	-3.444405513	-0.653612588	474.9303086	0.858517101	12.42516739	3.186305263	-13.17837862	-113.4500659	3.85E+07
Armenia	-0.019047619	-6103.785714	-0.219047619	0.764333333	11.08930894	4.933603838	315.7466153	0.603675918	-13.77081715	-2.707382456	23.42400226	234.9630563	3065398.517
Australia	-1.4	1348448.386	250.7809524	-7.623285714	4.043397257	-0.799819472	1283.260009	1.13446228	5.192544968	0.318252632	-4.925118173	56.76772159	2.05E+07
Austria	-0.433333333	-17189.28571	-21.14285714	-11.11428571	-6.12080791	-5.077310573	1458.773958	0.370438001	27.1905381	-3.934161404	16.80273986	301.045518	8209683.379
Azerbaijan	-0.380952381	-15085.71429	-45.27619048	36.25016667	8.699230741	-6.906679992	586.2318741	-0.645576503	-1.09736599	1.137989474	13.23239882	188.9515695	8476044.517
Bahamas	0.50952381	83676.19048	2.842857143	-11.83857143	14.20293477	6.015605356	404.6374246	-0.642691703	-14.7467787	2.176031579	7.823053025	96.53885338	321679.7931

Bangladesh	-3.00952381	-122871.4286	-18840.98095	-0.002	30.30951449	5.856454585	33.82991738	0.192670182	16.47678697	9.960852632	17.04187964	226.9015558	1.35E+08
Barbados	0.19047619	319.047619	0.2	7.084	-5.111606673	-0.040574847	428.7086055	1.238316691	-1.495005566	-4.065112281	-2.262240483	76.66646994	275103.7241
Belarus	-0.033333333	-11310.47619	346.5571429	29.56733333	6.681585588	3.442681212	464.7000903	0.412077454	7.037153564	7.896217544	34.76460989	477.7794191	9797946.207
Belgium	-0.171428571	-27070.21905	9.628571429	-18.15257143	2.480476842	-1.840701236	1406.705883	-1.402161053	35.06996612	1.019414035	17.06106164	219.3190528	1.06E+07
Belize	0.033333333	-36396.59048	-43.76666667	7.195285714	-6.635491478	3.469670715	165.3295523	-0.05295447	16.49949007	-1.331403509	1.144104558	30.10127973	278207.4828
Benin	-0.09047619	-236.7857143	23.55238095	0.860714286	1.150844558	-6.141803557	14.16789034	0.305981462	3.928041135	7.742312281	-2.896570963	-16.57321822	7920855.828
Bermuda	-0.071428571	-21428.57143	-0.285714286	-14.412	216.311229	0	0	-0.242896853	0	0	17.66895644	233.4800338	62718
Bhutan	-0.09047619	-250	-13.6047619	0	0	-13.12446992	81.25215919	0.396243633	26.39555998	15.46355789	2.344888915	45.06103134	631276.4138
Bolivia	0.047619048	29452.38095	-47.28571429	8.126142857	-1.902424637	0.179559563	122.3180453	0.505387548	24.30443548	8.830259649	4.675202401	39.78661764	9081172.448
Bosnia and Herzegovi	0.704761905	13619.61905	2.023809524	33.82433333	14.70145397	1.019790001	485.2016625	2.743593562	-16.94098032	5.407347368	90.51213438	946.2453141	3756990.31
Botswana	0.519047619	2576.190476	30.62380952	7.594	9.701548923	-3.426278788	285.4566663	0.50148739	-3.81046942	17.45163158	5.107383476	57.54998363	1770534.517
Brazil	0.214285714	821538.9524	104.5142857	18.62128571	1.148930176	2.913862799	641.9336131	0.496988495	6.525044174	7.928635088	8.745595225	88.60036231	1.82E+08
Brunei Darussalam	-0.071428571	-142.8571429	0	2.158571429	-1.306097378	-0.402769451	-193.5148189	3.571495396	-9.143995289	6.778270175	5.391585751	79.65051273	352380.5862

Bulgaria	1.142857143	92111.45714	19.10952381	19.94242857	15.19135372	2.624062556	573.7133403	-1.1560622	34.65477779	5.247308772	59.47516146	708.7670855	7812101.276
Burkina Faso	0.523809524	12078.4	244.6285714	-3.838571429	-3.490230445	-5.96560907	47.70739198	0.054464354	11.42173478	9.796831579	-0.985447006	-7.691683808	1.35E+07
Burundi	0.914285714	1000	-5.876190476	0	0	-4.348882011	23.34386025	-0.013181593	6.850794735	3.941238596	-4.242528064	-31.00664545	7618328.31
Cabo Verde	-0.023809524	73.33333333	-16.72857143	13.21214286	7.721549865	-17.36146843	101.1876976	0.647685932	18.62290281	15.32345614	4.63139517	56.95561278	450121.5172
Cambodia	0.047619048	39711.19048	-69.94761905	14.18228571	-4.814550265	-1.708242389	87.82954338	0.178505638	38.22475182	4.065238596	7.920310975	83.07904944	1.29E+07
Cameroon	-0.176190476	11.9047619	148.6	16.24757143	40.81059277	-1.570639577	36.13373084	0.010477302	7.846360731	10.18658596	-1.277888937	-1.211588487	1.77E+07
Canada	0.076190476	653159.5238	-13.70952381	-6.179	5.323385383	-0.595876031	1360.993563	-0.258483045	0.88784233	3.073217544	10.26052876	163.2898002	3.21E+07
Central African Repu	0.185714286	0	-26.22380952	-0.069142857	-0.698276055	-2.06623639	4.336957231	-0.01143695	-6.841137914	2.292578947	-0.160121479	10.51761141	3868991.483
Chad	0.861904762	595.2380952	219.1190476	1.720857143	2.629722241	-1.612052865	22.34775059	0.02312015	26.03878949	1.080119298	-4.847388343	-30.04277933	1.01E+07
Chile	0.30952381	2266904.762	771.9285714	14.26285714	-0.675810474	-0.461792244	608.3902841	1.400996094	9.79654452	2.696207018	10.78990601	112.7344126	1.60E+07
China	8.323809524	1.29E+07	-28665.32857	17.609	9.573655091	-0.528137788	277.9505442	3.579705612	17.26123747	19.92470526	61.62174559	566.361648	1.28E+09
Colombia	0.114285714	96785.54762	-580.1333333	10.54042857	3.679445174	0.951991217	266.3881671	-0.022969352	3.709892601	7.026645614	7.668480873	62.43127222	4.17E+07
Comoros	0.3809	333.33	8.5428	0	0	-1.498	-10.40	0.0516	1.9102	0.473421	0.98049	-3.63248	606629

(the)	52381	33333	57143			416099	292723	07519	80732	053	4987	9288	.1379
Congo	0.828571429	823.8095238	-112.8	7.428857143	0.251711292	-2.853878531	14.51920891	-0.032460851	26.35739783	8.176431579	-2.34439203	10.71400277	5.57E+07
Costa Rica	-0.261904762	-12706.42857	-29.82380952	2.429857143	-2.811587002	1.787615624	418.6091956	0.454433576	-4.235170393	18.70506316	8.334510403	97.30181378	4160821.69
Cote d'Ivoire	0.561904762	0	28.27142857	0	0	9.631214603	40.5985916	-0.104206438	12.92013765	6.850596491	0.318232886	18.90374673	1.81E+07
Croatia	0.557142857	-27344.04762	-64.5	-11.23166667	0.139528815	1.635608252	647.391494	0.600478659	12.86194196	3.499978947	54.14522563	446.7948146	4386398.862
Cuba	-0.476190476	1288897.981	-10.70952381	8.003571429	6.706729713	2.195470109	1305.388951	0.055910389	-8.08884644	2.698508772	25.05011749	245.1241422	1.11E+07
Cyprus	-0.438095238	-1024.285714	-41.4952381	-0.176285714	8.915365069	3.536567776	705.8024721	0.455046853	-0.03158137	0.05974386	-2.64324865	34.67706392	1001059.931
Czech Republic	0.628571429	-215065.8667	-197.1952381	0	0	-4.091343152	858.1803231	-1.43775119	53.66158375	-1.226866667	19.37697005	23.19319445	1.04E+07
Denmark	-0.095238095	-159998.0238	-0.242857143	-33.87242857	-3.590838651	-2.156977381	1651.459599	-2.804910184	27.46296506	1.977564912	-7.471821412	41.91207602	5428114.103
Djibouti	-0.176190476	-151.3571429	-12.56666667	0.309714286	4.740465567	-21.01431568	32.38378868	0.028344931	0	1.046396491	3.040361377	49.48358245	766029.4138
Dominica	0.119047619	116658.7619	16.18571429	-8.226571429	18.58533711	0	179.4987978	0.806411717	-3.339860461	5.265245614	12.32128452	125.9174786	70679.55172
Dominican Republic	1.652380952	-102419.4286	19.93809524	12.66871429	4.303661004	0.40486228	267.0957823	0.332074931	-11.0604679	16.37921404	10.55496753	105.5390868	8939247.517
Ecuador	-0.7	152039.881	338.8095238	15.195	-2.513975881	1.333808736	430.9627056	0.568284091	10.24858315	5.794407018	6.378039983	77.70390275	1.36E+07
Egypt	-0.338	-87523	-780.0	1.5847	8.9940	-1.709	93.832	0.7957	2.8926	-0.24604	12.6688	150.3146	7.52E+

	095238	.80952	857143	14286	8386	559775	39133	522	27619	9123	3765	832	07
El Salvador	0.152380952	-8432.619048	-700.3571429	6.764571429	3.921006421	11.02483185	87.20085208	0.315900032	19.94586978	13.04111579	5.549558403	50.23742212	5958408.966
Equatorial Guinea	0.066666667	0	1	0	0	-34.27545643	192.9684785	4.20034977	0	25.43950877	-7.349664267	-34.23731559	774602.7586
Eritrea	-0.223809524	-368.9285714	-0.49047619	0	0	-1.023839638	-11.91706342	-0.128495358	-49.3633957	11.20011842	-1.498623468	-8.441237634	2548038.318
Estonia	0.266666667	8666.666667	0.733333333	64.016	7.010571239	-1.316273062	830.14382	0.966438772	9.40671834	-1.917235088	5.263051983	-221.6490469	1384261.759
Eswatini	0	0	0	-10.60014286	22.04562864	0.563263077	284.8000771	0.288213515	-33.61744536	1.440122807	0	0	1011059.345
Ethiopia	0.00952381	92516.20952	41.2	4.245272727	10.21748261	-0.750422912	23.53228279	0.028575229	0	4.368603509	-1.044373269	-5.557012021	7.58E+07
Fiji	0.614285714	45742.20952	11.53333333	5.797571429	20.95185533	-1.09652396	83.0248303	0.32963629	0	8.502568421	14.35151688	169.3279753	819626.6552
Finland	-0.076190476	-714.2857143	0	2.411142857	3.434386047	-3.825496403	1378.364234	-0.279178842	18.23391964	4.003312281	37.54245721	431.6633506	5255677.414
France	-1.314285714	-845271.4286	-1095.666667	-20.27171429	-1.042871828	1.262518665	1255.214669	-0.809197931	11.97865619	3.782175439	14.26183121	217.7299787	6.27E+07
French Polynesia	-0.00952381	866.6666667	-0.647619048	3.739285714	1.37338792	6.634599607	0	0.632296561	0	3.092582456	0	0	247328.9655
Gabon	0.319047619	0	-8.080952381	1.121285714	-1.159126989	-11.76547976	-9.236250653	-1.674045351	-6.999782795	12.95653684	-2.524991898	5.151701706	1429358.276
Gambia	0.366666667	0	-6.833333333	5.954714286	1.692674259	2.492465886	35.35301591	0.031995792	-9.042078443	14.21432632	2.145475339	32.00288316	1539153.621
Georgia	0.2904	-33606	-15.30	8.9613	-8.828	-2.292	332.98	0.5027	16.304	2.023838	30.1814	361.4403	410463

	7619	.26667	952381	33333	625787	028383	94539	63771	45723	596	0994	108	8.724
Germany	0.023809524	251003.3333	-645.9142857	-21.20571429	7.625811378	-5.091706534	1417.099207	-1.16002727	31.42777955	2.932982456	20.69286832	292.9397437	8.17E+07
Ghana	0.461904762	-1592.857143	2033.304762	-3.164428571	19.26807824	-1.559723412	61.30140886	0.115232763	11.29500606	11.94869825	3.146447979	54.33332824	2.17E+07
Greece	-0.766666667	-382432.2571	-147.252381	0.871428571	-36.65510865	4.592202506	683.8672291	0.41182156	16.19312347	4.63665614	16.1162186	251.0638728	1.08E+07
Grenada	-0.00952381	58873.80952	2.6	3.345428571	-0.625117049	-7.462512191	204.9947041	0.950951808	-1.943235776	1.554866667	15.31171762	207.9256601	104092.3793
Guam	-0.223809524	-53504.7619	-45.29047619	0	0	11.20801901	0	0	7.66886655	2.037280702	23.4231045	311.4118665	154061.9655
Guatemala	1.380952381	115524.2762	1175.457143	1.570857143	2.340803898	1.277780451	100.8625489	0.241018937	13.79854474	5.420522807	-0.062356135	4.39187541	1.30E+07
Guinea	0.685714286	0	428.1571429	0.256428571	-8.386331677	0.475395508	0.173598546	0.043968345	27.79623287	4.822470175	-4.146329272	-25.27727251	9083260.241
Guinea-Bissau	-0.038095238	0	-125.3619048	2.387142857	-0.236957201	-1.93636255	20.22271394	-0.016563714	0.04579541	7.937747368	-3.492233206	-22.55535492	1357397.448
Guyana	0.257142857	41181.90476	2.266666667	0.292857143	0.890629593	-4.035722523	93.0778475	0.394148157	-54.29146233	-2.348140351	3.807374097	97.2536157	754020.3103
Haiti	2.89047619	679724.7619	55368.35238	1.595428571	-4.554381435	-1.074458174	20.49885058	0.089628564	24.10304499	16.9014807	-2.338435901	-27.453714	9065537.448
Honduras	0.4	-277203.781	-1910.604762	2.505142857	1.050928942	-1.769842928	149.0300721	0.382339607	26.06451405	10.07550526	4.230386496	23.17587737	7267610.345
Hungary	0.276190476	-29471.42857	82.94761905	-10.40414286	-0.534259442	2.463621296	606.4494267	-1.284218794	64.77012373	3.734389474	2.474804512	3.333211446	1.01E+07
Iceland	-0.290	-3306.	-3.928	37.398	3.7986	-1.229	668.94	-0.832	20.461	2.125364	8.02488	101.1706	297165

	47619	357143	571429	57143	1453	262895	96801	420866	39002	912	3283	075	.5517
India	2.8	2501064.943	-16122.80952	0.315571429	24.62729659	-0.430773933	67.02866495	0.552215644	26.3517567	4.952431579	5.852224384	86.02366586	1.12E+09
Indonesia	4.147619048	487448.9952	4679.061905	0.214857143	14.5454216	-2.49115963	137.5922237	0.782402117	-6.798661374	15.28998596	15.05560439	147.1799736	2.24E+08
Iran	-2.195238095	-1088556.581	-13563.25238	0.008857143	8.094261942	3.328245146	486.5705717	3.135775654	12.38332132	11.52854737	25.52853586	194.0322888	6.90E+07
Iraq	0.714285714	86.66666667	190.5714286	-0.759142857	-4.326352135	-0.969965154	199.8453688	0.674169258	21.52407767	0.360985965	3.054847505	13.93450732	2.66E+07
Ireland	-0.395238095	-3551.190476	-1.028571429	-11.22928571	-16.27080166	0.404961339	2118.791696	-0.421589543	53.63564454	3.859898246	-8.311833448	-26.51167384	4132010.931
Israel	-0.242857143	1755.952381	222.7095238	3.971428571	-1.978223147	-0.495191333	378.0204186	0.426707257	4.190804858	1.213063158	8.670186033	82.22733538	6807389.655
Italy	0.19047619	-190292.9333	-1305.014286	-15.10671429	-12.39312039	0.108788304	839.8665521	-0.354174636	12.74619053	1.779887719	-8.904163322	-9.997142012	5.82E+07
Jamaica	0.352380952	88374.30952	3.133333333	-0.658142857	-0.182212354	-1.162301405	78.16909596	-0.026491775	-8.090774093	3.612814035	4.294444821	38.54707036	2703245.103
Japan	2.185714286	1.21E+07	10765.31905	-22.56242857	-1.687530443	2.599932389	1348.664659	0.577536799	12.30076642	11.46839649	24.4230257	396.264126	1.27E+08
Jordan	-0.50952381	-28642.85714	-13.32380952	-0.546714286	1.071817369	11.37320495	110.2773508	0.223372164	1.356699224	10.92775439	10.49947033	80.83625108	6248447.793
Kazakhstan	-0.052380952	13371.67619	-1.147619048	22.00316667	9.642756157	-0.127873827	257.4306013	3.821326753	-13.02152133	0.788305263	9.627222216	170.7979176	1.61E+07
Kenya	1.828571429	38254.42857	346.1761905	-0.201714286	2.142568348	0.459396424	42.88132596	0.019682853	-4.744664739	5.92582807	0.900675939	11.85467538	3.64E+07
Kiribati	0.1285	0	0	-1.802	0.5799	2.2698	20.556	0.2228	-9.296	10.64981	3.73963	41.54713	92383.

	71429			714286	5679	45686	35202	4538	533791	754	1766	937	44828
Korea, Dem. People'	0.8	-1639381.381	164.4333333	-2.445285714	-7.561976883	-1.394337077	0	-0.425422929	0	1.849101754	32.14620398	250.4512331	2.34E+07
Korea, Rep.	-0.652380952	-759103.7619	-107.0714286	7.173857143	0.55170535	0.518457427	973.8548311	3.443001172	30.9192024	6.037217544	28.73657197	392.8718066	4.78E+07
Kuwait	-0.076190476	0	-0.076190476	0.194714286	0.250163623	-5.547166879	333.4426941	6.154596493	0.96737432	1.521578947	15.20664753	134.9554289	2647571.5
Kyrgyzstan	0.480952381	-13502.19048	6.59047619	4.375166667	-16.84999427	-1.357386862	123.2522334	0.094741035	38.7351578	-1.165007018	-9.187790293	-133.867383	5169151.724
Lao PDR	-0.295238095	-8152.309524	-53.14285714	3.345142857	21.2113553	0.174729092	21.88016425	0.135684838	31.37399574	12.65155088	0.260365919	13.22623112	5680999.31
Latvia	-0.019047619	21630.95238	0.333333333	41.175	1.411682184	2.979531676	576.2265884	-0.08804439	25.94948975	-0.687392982	81.41623729	711.5621595	2266691.034
Lebanon	0.119047619	-11071.42857	45.25714286	1.248428571	-87.59186007	-7.869311901	116.3898667	0.111960801	16.57992462	3.435849123	10.66026045	85.73579516	4594556.897
Lesotho	-0.176190476	0	-3.980952381	3.136428571	-0.606721362	-9.151895196	95.23016435	0.200507192	0	8.822178947	5.255124134	72.44302338	1973209.897
Liberia	-0.257142857	-3357.142857	1025.071429	-0.433571429	-7.556009447	-3.075793323	66.22231007	-0.003969138	99.70557529	0.573329825	-6.810205172	-66.36787483	3223205.966
Libya	-0.004761905	-3014.285714	1.066666667	0	0	12.08721392	-97.21930523	0.146617548	47.95547363	2.372778947	18.00205231	138.4745962	5652290.69
Lithuania	0.30952381	12895.34286	-1.352380952	60.34966667	1.334890757	8.452798513	748.7439478	0.042358181	47.6528197	-0.015196491	92.78544608	1001.735157	3314005.31
Luxembourg	-0.576190476	-25790.47619	-12.14285714	17.67028571	-4.916848143	-7.093982543	2048.170503	-1.073048954	142.172135	6.316217544	-14.77186515	-98.82563116	472705.2759
Madagascar	1.1571	33813.	436.50	1.112	2.4466	-0.009	8.6094	0.0035	26.486	7.640270	-4.5204	-47.7381	1.82E+

	42857	2619	47619		78354	183297	21696	92604	51159	175	95758	679	07
Malawi	0.480952381	23729.35714	-39.82380952	3.953571429	4.662050005	2.336856119	49.74268028	-0.006757171	2.446484631	2.979094737	-1.760771157	-9.142418088	1.28E+07
Malaysia	0.038095238	102438.2286	-1.90952381	0.885428571	3.497102324	-5.177077326	340.7493632	2.912470444	-6.085392838	16.4625614	8.2345839	127.6150386	2.50E+07
Maldives	0.128571429	29197.14286	154.6666667	1.666571429	6.944021666	-59.69491141	431.4025763	1.471543737	3.001263348	10.0630807	3.050392092	43.4469261	330657.4138
Mali	0.733333333	0	-96.44761905	0.269	0.627434318	-4.320702524	22.87045338	0.01182958	7.032557741	11.4767193	-5.692703822	-53.25332428	1.29E+07
Malta	0	0	0	2.858	4.506907211	-3.519602965	1079.500585	0.383900194	87.54933769	2.968	5.860710892	143.5376117	404297.9655
Marshall Islands	0.123809524	326.6666667	-0.428571429	0	0	0	212.0416587	0.412773682	0	7.639666667	8.291156357	76.06284788	53604.75862
Mauritania	0.071428571	133.3333333	-19.07619048	-0.015428571	10.80104563	-3.683990944	20.61894488	-0.011606356	21.77888413	8.895073684	-4.535041419	-36.52655146	3043990.966
Mauritius	-0.161904762	-25609.52381	-69.23809524	0.859	1.920116612	1.979011167	415.6700549	1.420990034	-13.97951806	-1.606929825	12.32200451	120.7517755	1197033.724
Mexico	-0.8	1855835	146.4047619	6.802285714	0.745537127	2.666334071	309.5247446	0.317560045	23.96997833	5.403347368	6.098494007	57.37942935	1.05E+08
Moldova	-0.104761905	-18.81904762	-8.342857143	0	0	-2.258227209	281.2357226	-1.020221468	-18.54143308	-2.926901754	41.02921694	281.1150904	3613634.31
Mongolia	-0.476190476	-132508.2571	-10.43809524	16.27342857	-2.07971142	0.991256112	103.6434524	2.034586144	11.29574529	9.433396491	5.727139709	100.3800496	2570941.345
Montenegro	0.4	0	0.066666667	0	0	2.375593533	0	0	18.14750475	12.28886667	36.9771703	368.8089281	614136.7586
Morocco	0.4047	-34754	43.566	-1.093	6.0330	2.4503	183.70	0.6003	25.000	8.174961	22.6291	202.4542	3.03E+

	61905	.45238	66667	428571	66756	3159	43374	03446	88763	404	3927	241	07
Mozambique	0.1428<br>57143	-21575<br>.47619	168.23<br>80952	1.6512<br>85714	-59.63<br>475463	-5.300<br>777526	23.546<br>1246	0.0544<br>72513	41.639<br>81187	6.530010<br>526	-3.1232<br>87701	-16.0016<br>3172	2.04E+<br>07
Myanmar	2.0190<br>47619	302963<br>.2714	10666.<br>6381	0.998	1.4973<br>7414	3.0709<br>33583	83.259<br>54727	0.1016<br>58888	16.209<br>52925	3.144042<br>105	8.57499<br>4946	96.94579<br>008	4.80E+<br>07
Namibia	0.5571<br>42857	5989.8<br>09524	44.152<br>38095	49.864<br>14286	2.9176<br>06389	-1.242<br>077952	291.56<br>81913	0.4540<br>99107	5.8251<br>71507	13.04047<br>368	-2.9220<br>19318	-23.4945<br>8234	192607<br>8.138
Nepal	0.6857<br>14286	373501<br>.0857	1613.0<br>57143	0.7458<br>57143	42.465<br>81413	20.736<br>44542	45.389<br>17497	0.0862<br>35639	2.1009<br>13562	6.483849<br>123	7.40368<br>9897	82.61961<br>101	2.46E+<br>07
Netherlands	-0.280<br>952381	-16501<br>1.1714	-8.542<br>857143	-15.87<br>1	-1.257<br>241973	-1.494<br>329364	1953.4<br>85227	-0.229<br>802805	31.156<br>07461	14.94992<br>281	7.52371<br>3108	150.0972<br>331	1.62E+<br>07
New<br>Caledonia	-0.214<br>285714	-2857.<br>142857	-7.285<br>714286	-0.727<br>285714	3.5477<br>07747	4.7176<br>86896	0	3.4042<br>14287	0	6.857024<br>561	0	0	228190<br>.6552
New Zealand	-0.090<br>47619	197048<br>7.619	119.57<br>14286	-20.63<br>342857	-4.041<br>65528	0.6553<br>22972	1159.7<br>49655	0.0830<br>93518	-0.129<br>021663	1.089157<br>895	11.7275<br>5609	125.6627<br>896	408119<br>3.103
Nicaragua	-0.433<br>333333	-80162<br>.28571	-274.2<br>714286	3.4254<br>28571	0.6938<br>85387	2.0842<br>14383	151.34<br>30994	0.1745<br>25155	37.237<br>81859	3.188147<br>368	4.55710<br>4121	45.21637<br>018	535479<br>4.552
Niger	0.9285<br>71429	17389.<br>26667	253.53<br>33333	0.0562<br>85714	19.591<br>1461	-6.793<br>056546	16.690<br>34999	-0.021<br>565825	15.112<br>26047	0.617901<br>754	-3.0186<br>24382	-29.0888<br>1381	1.39E+<br>07
Nigeria	0.3619<br>04762	50083.<br>01429	945.00<br>47619	1.6057<br>14286	12.003<br>03885	-2.113<br>308749	103.63<br>81562	0.0880<br>01014	1.3715<br>41293	12.74065<br>263	-5.3898<br>10395	-32.7579<br>1791	1.39E+<br>08
North<br>Macedonia	0.4285<br>71429	-8428.<br>785714	15.052<br>38095	-10.48<br>366667	2.9404<br>94997	1.5711<br>34877	190.98<br>13115	-1.066<br>496597	28.886<br>18678	-0.80001<br>0526	42.3737<br>2461	397.7188<br>175	204176<br>8.414
Northern<br>Mariana Isl	0.0571<br>42857	0	1	0	0	0	0	0	-25.72<br>943219	1.395866<br>667	25.2411<br>4789	283.3872<br>471	53301.<br>34483
Norway	-0.3	-22261	6.8619	10.046	4.8526	-5.705	1987.5	1.3813	-0.366	5.942498	20.4499	287.7481	468479

		.90476	04762	42857	17666	577548	64446	94096	225683	246	7453	241	8.724
Oman	0.39047619	337757.1429	16.13333333	1.150571429	5.750203151	-49.0393769	171.645943	7.54578174	21.80170863	9.174424561	5.185851895	38.65916834	2828882.793
Pakistan	0.928571429	1593105.771	14552.10476	-0.137857143	-0.571766638	1.728549609	31.42214379	0.220119657	-3.247608439	3.664687719	-2.696804482	8.370955066	1.58E+08
Palestine	0.466666667	0	7.333333333	0	0		0	0	0	0	1.09560861	37.45913593	
Panama	0.376190476	18053.57143	-38.52380952	19.19371429	1.306237382	2.166865662	527.8825598	0.751854985	4.22350647	8.025396491	6.114904567	95.74193083	3286944.793
Papua New Guinea	0.2	-1721.047619	-216.9571429	0	0	-0.917920399	5.754975221	0.244103899	29.11868186	-0.969989474	0.790868926	26.92664815	6468297.69
Paraguay	1.20952381	3643.690476	3.119047619	-9.375571429	0.603825293	-1.388936776	225.0523934	0.110710774	-14.50390935	8.209817544	4.875771243	47.49071958	5663529.448
Peru	-0.123809524	213300.9524	114963.5619	9.165285714	191.641339	0.330008134	201.1155456	0.602490394	17.7403962	5.963189474	8.348704705	82.62049859	2.73E+07
Philippines	4.957142857	1089932.89	10815.4619	-0.026571429	0.199901664	0.65579239	130.3706456	0.118623115	-2.446316886	0.048785965	12.48815146	115.6411548	8.44E+07
Poland	0.995238095	-63641.19048	36.9047619	40.73485714	-0.16075626	1.894565687	631.106211	-1.460390192	31.61436106	-0.535484211	42.73435702	212.2131014	3.82E+07
Portugal	0.676190476	247175.7333	-114.1904762	-14.50671429	1.025579225	4.183477396	757.3619267	0.304867392	12.3026971	10.5466807	-20.15775587	-88.53753627	1.03E+07
Puerto Rico	0.019047619	4355619.048	1.733333333	0	0	2.401298944	0	0	6.777062772	1.416042105	20.66100699	310.8698776	3664717.276
Qatar	0.066666667	0	0	0	0	-94.80453552	364.997403	-2.666322638	6.349762648	4.279350877	2.177863639	-31.41532975	1250105.724
Romania	0.5619	83406.	10.461	29.670	40.294	4.2014	530.13	-1.914	19.719	0.881880	63.6392	504.8930	2.14E+

	04762	39048	90476	14286	61788	49352	68566	264099	74047	702	0473	34	07
Russian Federation	-3.771428571	346687.5476	3885.985714	46.19933333	4.873623162	4.323114498	620.5703411	0.60913667	-6.946242148	0.611547368	15.76273784	340.6962018	1.45E+08
Rwanda	0.123809524	1867.266667	48.14285714	-1.680285714	-5.379886445	-1.538620809	72.26472132	-0.014511429	13.44067729	7.269919298	-3.358908691	-11.98703711	8772451.828
Samoa	0.185714286	-17114	30.5	1.541	2.705880901	3.659866915	130.0797615	0.248380539	-1.435767566	-1.667238596	9.949556637	92.15875859	179503.6897
Sao Tome and Princip	0.066666667	0	1.666666667	6.941	7.085664276	-4.652108693	-6.152603121	0.140878326	0	18.29278246	-3.399637701	-12.94584365	159803.6897
Saudi Arabia	0.638095238	83466.66667	10.34761905	-0.649857143	-2.424286173	-13.26845064	556.2679776	3.831736225	13.74416066	4.560621053	7.901305904	86.59741505	2.40E+07
Senegal	0.271428571	-2260.404762	-14.14761905	-0.976857143	12.22767204	6.811203675	29.53164202	0.104258261	-0.731902018	4.798792982	0.299180709	13.15155627	1.12E+07
Serbia	1.6	152034.8	47.73333333	-7.658333333	-3.226181451	2.030289696	657.4837324	0	41.38623292	3.054190476	50.83197647	651.5003416	7405198.828
Seychelles	0.19047619	2498.571429	16.56666667	0	0	-12.3120594	74.43231101	2.459190342	100.2329573	4.067	4.618875364	58.21314026	83139.41379
Sierra Leone	0.004761905	2000	1294.366667	-0.897142857	2.505059848	-2.546551698	71.38778989	0.015000386	16.52085308	5.069045614	-6.854300603	-42.34855205	5568030.345
Singapore	-0.147619048	0	867.4952381	0	0	-10.21668597	1027.613632	-4.885480333	37.33701318	0	7.461633314	149.386911	4398745.069
Slovakia	0.366666667	270	7.876190476	-9.271090909	-0.673841237	0.987356071	1045.633481	-0.91224476	59.17829792	-1.47765614	45.39529242	366.672508	5380374.828
Slovenia	0.323809524	39085.71429	-19.57619048	-16.3395	36.08926135	-7.544933557	794.9647526	0.224087149	29.38747473	2.418273684	8.607719845	62.08285702	2017309.207
Solomon	0.6333	2000	90.533	6.5625	4.7698	4.8290	47.103	-0.077	17.882	5.980410	3.73553	67.98260	465071

Islands	33333		33333	71429	83825	9719	16927	044509	4111	526	8663	937	.7241
Somalia	1.133333333	6801.333333	3029.690476	0	0	1.887104712	0	-0.039136092	85.09738636	8.87614386	-2.563880704	-33.90261387	1.04E+07
South Africa	-1.09047619	53929.43333	-92.58571429	-7.864571429	-0.201467495	0.58999307	282.5369168	0.486018574	12.96072077	8.761007018	8.680658662	103.2171654	4.74E+07
South Sudan	1.2	0	323.6	0	0	-2.130064741	0	0	0	3.579333333	-4.101848086	-29.4970205	7682151.552
Spain	-0.080952381	-336900.9048	-1076.07619	-3.054571429	10.77974636	-2.382045155	1115.027627	0.471410314	13.79582787	3.022992982	1.440637659	136.7870106	4.31E+07
Sri Lanka	1.204761905	241887.1429	14431.02857	2.387	2.489681427	14.61593591	134.7200144	0.334233572	-16.06438243	-0.196129825	15.8776903	164.0995941	1.94E+07
St. Kitts and Nevis	-0.219047619	-44266.66667	-0.357142857	-2.768285714	-0.199342894	0	394.2275011	1.629412672	-6.927303705	-2.551508772	0	0	46198.62069
St. Lucia	0.319047619	2733.333333	1.114285714	-5.247857143	-14.55157774	-1.218684578	144.4001223	0.598060985	-14.2396236	-8.531473684	11.11899665	114.6737625	162148.0345
St. Vincent and the	0.319047619	8847.619048	3.033333333	15.63642857	4.691674054	-6.438151023	110.6864195	1.008678341	-17.58889261	6.573315789	16.39680379	177.261405	108376.6897
Sudan	0.166666667	4309.52381	757.9666667	-3.099464286	11.12210431	1.901639603	140.9790323	0.062893294	12.72792528	3.313550877	1.65005878	20.04780779	3.05E+07
Suriname	0.133333333	0	0.333333333	10.71714286	-0.246233976	3.061767414	159.986789	-0.862252941	0	0.31417193	10.18760294	136.160444	493641.3448
Swaziland	0.038095238	-127.7857143	-9.20952381	0	0		0	0	0	0	4.699559536	71.83120379	
Sweden	-0.161904762	177523.8095	0.647619048	-8.551571429	3.78078537	-4.005193445	1640.228728	-1.059371229	19.88009983	1.957364912	-12.87591887	-1.61073242	9158409.793
Switzerland	-0.3	-39180	-71.20	-7.565	3.1533	-4.365	2059.3	-0.838	28.146	-0.10797	5.16605	98.08869	750709

		.47619	47619		59116	599985	47279	876825	81576	193	8523	877	6.241
Syrian Arab Republic	0.119047619	0	225.3619048	0	0	0.589701127	6.677425601	-0.367972027	19.68762181	3.629852632	7.691905602	66.01076669	1.71E+07
Tajikistan	0.180952381	28232.28571	-105.547619	-1.879166667	8.489243028	-4.734218664	80.46477147	-0.108398484	-43.05826479	-2.920182456	-0.672150799	10.59411181	6868517.655
Tanzania	-0.19047619	30395.95238	-837.6428571	-0.694142857	-54.35906385	-1.276596925	41.29373474	0.072439084	1.326555243	8.664049123	0.524768067	9.401332002	3.87E+07
Thailand	-0.228571429	2981448.138	1047.552381	15.97985714	19.61601338	5.333705092	207.5208471	1.838098503	44.44025096	13.48968772	17.94536468	224.546197	6.41E+07
Timor-Leste	0.319047619	266.6666667	14.71428571	-1.646857143	0.523274707	-0.467290352	69.18119414	0.062833275	-24.42239094	5.984835088	13.59751769	124.9633821	986741.7586
Togo	-0.123809524	0	-26.36190476	-0.878142857	-3.848681281	-3.057231738	29.81958086	0.093713995	11.56709315	7.784326316	1.977022527	25.60740518	5590454.621
Tonga	0.242857143	-942.8571429	1.780952381	0	0	6.682084125	101.9537261	0.27445599	-0.240743856	0.463094737	9.350898	68.86998784	99597.65517
Trinidad and Tobago	-0.095238095	-1481.452381	-0.3	2.083714286	0.940009857	2.84442696	787.8168652	16.78810433	0	-0.759417544	8.271816926	191.5968702	1298511.586
Tunisia	0.185714286	-14942.85714	0.1	1.349714286	15.75587642	6.131102752	274.5677551	0.593948701	15.19324721	6.806410526	32.02564305	237.921169	1.00E+07
Turkey	-0.428571429	-1507811.905	-5143.528571	2.087571429	1.408519486	0.782966878	396.0501033	1.161057999	10.58520171	10.42506667	22.74294194	208.775082	6.72E+07
Turkmenistan	-0.142857143	-7133.571429	-0.785714286	2.037	3.625085982	0.013424272	291.718165	3.229215999	-31.18843969	3.523080702	10.78153264	95.265134	4753618
Uganda	0.371428571	-4794.314286	86.66666667	-45.89485714	-67.83720125	2.181824144	61.58164559	0.05769031	15.88518113	7.459705263	-3.019984809	-15.9494437	2.79E+07
Ukraine	-0.014	152484	832.80	42.692	6.8743	3.0366	241.66	-1.446	17.499	1.906638	2.32364	293.9768	4.81E+

	285714	.2143	95238		17162	65579	99951	768604	45821	596	6844	895	07
United Arab Emirates	0.0666<br>66667	0	0	0	11.044<br>52251	-49.86<br>727807	121.50<br>91609	-6.927<br>923882	56.446<br>60939	4.882922<br>807	3.23817<br>781	58.40980<br>127	535322<br>3.103
United Kingdom	-0.666<br>666667	17089.<br>52381	19.557<br>14286	-12.69<br>671429	7.4790<br>17241	-4.820<br>851161	1327.5<br>66706	-1.562<br>47215	8.0761<br>26131	3.099371<br>93	-12.522<br>17535	-17.4771<br>2775	6.09E+<br>07
United States	-4.5	4.00E+<br>07	-258.4<br>333333	-4.924<br>285714	-4.548<br>891284	-2.403<br>035538	2813.3<br>02955	-1.589<br>854573	6.9199<br>0766	4.037785<br>965	1.17377<br>8425	157.3995<br>664	2.92E+<br>08
Uruguay	-0.133<br>333333	16533.<br>33333	18.076<br>19048	-6.722<br>142857	-0.877<br>046202	1.9748<br>91028	416.54<br>44955	0.6287<br>51742	12.122<br>57217	4.252063<br>158	-5.8071<br>13256	-25.2968<br>1628	331103<br>0.931
Uzbekistan	-0.223<br>809524	-3571.<br>428571	-4.419<br>047619	2.8176<br>66667	23.428<br>1494	-0.832<br>890433	124.60<br>17111	-0.820<br>590476	6.2960<br>66092	6.719677<br>193	3.82013<br>3201	78.81470<br>81	2.63E+<br>07
Vanuatu	-0.280<br>952381	29664.<br>7619	6.5	-2.614<br>571429	-4.038<br>115195	4.3205<br>28296	23.002<br>3237	-0.033<br>530619	-1.090<br>254246	4.516249<br>123	6.27069<br>0357	78.58519<br>252	210213<br>.5517
Venezuela	-0.023<br>809524	-21003<br>5.7143	-2443.<br>452381	4.9425<br>71429	-0.610<br>096485	4.2210<br>68514	350.27<br>73566	-0.010<br>99807	3.2944<br>78886	2.6178	6.50613<br>3163	67.52506<br>781	2.56E+<br>07
Vietnam	1.7619<br>04762	970249<br>.1238	4915.1<br>04762	8.2452<br>85714	1.9700<br>36855	-2.219<br>949196	139.45<br>88832	0.9598<br>95658	81.759<br>03233	9.094105<br>263	9.49813<br>3801	134.5915<br>757	8.28E+<br>07
Virgin Island (U.S.)	-0.085<br>714286	-86857<br>.14286	-0.247<br>619048	0	0	5.6425<br>40936	0	0	77.442<br>41514	5.161445<br>614	27.5707<br>2667	351.2500<br>152	107746<br>.8966
Yemen	0.5190<br>47619	-46535<br>.71429	48.6	-0.651<br>857143	2.8090<br>24901	-0.834<br>014168	41.138<br>14281	0.1582<br>29075	0	9.314540<br>351	5.19102<br>6239	69.52489<br>896	1.99E+<br>07
Zambia	0.0476<br>19048	-1478.<br>571429	256.80<br>47619	-8.404<br>714286	1.9596<br>18209	2.0488<br>52711	17.623<br>04679	-0.064<br>095041	7.9406<br>05128	2.403814<br>035	-0.9917<br>06919	-0.36456<br>3561	1.20E+<br>07
Zimbabwe	0.2380<br>95238	24025.<br>2381	549.81<br>90476	6.8457<br>14286	0.9822<br>76099	15.373<br>96927	0	-0.605<br>850733	7.6602<br>87066	2.285294<br>737	2.03941<br>1365	32.53266<br>68	1.23E+<br>07
