# Supplementary material for: Changes in the incidence and prevalence of ischemic stroke and associations with natural disasters: an ecological study in 193 countries
Source: Sci Rep. 2022 Feb 2;12:1808. doi: 10.1038/s41598-022-05288-7 (PMC8810883; doi:10.1038/s41598-022-05288-7)
Supplement: Supplementary file 3 — Supplementary Tables. [file 41598_2022_5288_MOESM3_ESM.docx]

**Table 1.** Multivariable linear regression-derived coefficients for natural disaster and socioeconomic variables for the incidence and prevalence of ischemic stroke in females from 1990 to 2017 in 193 countries, weighted by population.

|  | Incidence^*^ | | | Prevalence^†^ | | |
| --- | --- | --- | --- | --- | --- | --- |
| Factors | Coefficient (95% CI) | Standardized Coefficients | P value | Coefficient (95% CI) | Standardized Coefficient | P value |
| Fat & meat consumption | NA^‡^ | 0.011 | 0.779 | NA^‡^ | 0.009 | 0.830 |
| Tobacco use  Alcohol consumption | NA^‡^ | 0.068 | 0.084 | 4.460±2.122 | 0.084 | 0.037 |
|  | 0.348±0.070 | 0.215 | 0.000 | 4.272±0.665 | 0.285 | 0.000 |
| Health expenditure | 0.005±0.001 | 0.149 | 0.000 | 0.096±0.014 | 0.303 | 0.000 |
| CO_2_ emissions | 6.228±0.895 | 0.460 | 0.000 | 58.216±8.459 | 0.464 | 0.000 |
| Trade (% of GDP) | 0.146±0.055 | 0.103 | 0.008 | 1.224±0.521 | 0.093 | 0.020 |
| Urban population (% of total) | 1.178±0.198 | 0.360 | 0.000 | 9.545±1.886 | 0.315 | 0.000 |

^*^ The stepwise method was used, R^2^=0.732, F=101.275, p=0.000

^†^ The stepwise method was used, R^2^=0.723, F=79.921, p=0.000

^‡^ Not applicable for variables not included as predictors.

**Table 2.** Multivariable linear regression-derived coefficients for socioeconomic variables for the incidence and prevalence of ischemic stroke in males from 1990 to 2017 in 193 countries, weighted by population.

|  | Incidence^*^ | | | Prevalence^†^ | | |
| --- | --- | --- | --- | --- | --- | --- |
| Factors | Coefficient (95% CI) | Standardized Coefficient | P value | Coefficient (95% CI) | Standardized Coefficient | P value |
| Fat & meat consumption | NA^‡^ | 0.005 | 0.906 | NA^‡^ | 0.025 | 0.534 |
| Tobacco use  Alcohol consumption | NA^‡^ | 0.031 | 0.420 | NA^‡^ | 0.062 | 0.121 |
|  | 0.449±0.078 | 0.238 | 0.000 | 3.746±0.689 | 0.235 | 0.000 |
| Health expenditure | 0.010±0.002 | 0.242 | 0.000 | 0.142±0.014 | 0.419 | 0.000 |
| CO_2_ emissions | 7.133±1.003 | 0.451 | 0.000 | 62.178±8.862 | 0.466 | 0.000 |
| Trade (% of GDP) | 0.166±0.061 | 0.100 | 0.07 | 1.524±0.542 | 0.109 | 0.005 |
| Urban population (% of total) | 1.456±0.222 | 0.381 | 0.000 | 10.977±1.966 | 0.340 | 0.000 |

^*^ The stepwise method was used, R^2^=0.754, F=113.102, p=0.000

^†^ The stepwise method was used, R^2^=0.729, F=99.515, p=0.000

^‡^ Not applicable for variables not included as predictors.

**Table 3.** Multivariable linear regression-derived coefficients for socioeconomic variables for the incidence and prevalence of ischemic stroke in both sexes from 1990 to 2017 in 193 countries, weighted by population.

|  | Incidence^*^ | | | Prevalence^†^ | | |
| --- | --- | --- | --- | --- | --- | --- |
| Factors | Coefficient (95% CI) | Standardized Coefficient | P value | Coefficient (95% CI) | Standardized Coefficient | P value |
| Fat & meat consumption | NA | 0.007 | 0.846 | NA | 0.019 | 0.640 |
| Tobacco use  Alcohol consumption | NA | 0.047 | 0.213 | NA | 0.074 | 0.062 |
|  | 0.399±0.073 | 0.228 | 0.000 | 4.159±0.668 | 0.270 | 0.000 |
| Health expenditure | 0.007±0.001 | 0.199 | 0.000 | 0.117±0.014 | 0.358 | 0.000 |
| CO_2_ emissions | 6.703±0.933 | 0.458 | 0.000 | 60.406±8.588 | 0.469 | 0.000 |
| Trade (% of GDP) | 0.156±0.057 | 0.102 | 0.007 | 1.312±0.525 | 0.097 | 0.013 |
| Urban population (% of total) | 1.321±0.207 | 0.373 | 0.000 | 10.044±1.905 | 0.323 | 0.000 |

^*^ The stepwise method was used, R^2^=0.751, F=111.429, p=0.000

^†^ The stepwise method was used, R^2^=0.727, F=98.57, p=0.000

^‡^ Not applicable for variables not included as predictors.
